# Dysregulated CD25 and Cytokine Expression by γδ T Cells of Systemic Sclerosis Patients Stimulated With Cardiolipin and Zoledronate

**DOI:** 10.3389/fimmu.2018.00753

**Published:** 2018-04-13

**Authors:** Helena Migalovich Sheikhet, Jose Villacorta Hidalgo, Paul Fisch, Alexandra Balbir-Gurman, Yolanda Braun-Moscovici, Ilan Bank

**Affiliations:** ^1^Laboratory of Immune-Regulation, Sheba Medical Center, Ramat Gan, Israel; ^2^Department of Clinical Pathology, University of Freiburg Medical Center, Freiburg, Germany; ^3^B. Shine Rheumatology Unit, Rambam Health Care Campus and The Ruth and Bruce Rappaport Faculty of Medicine, Technion – Israel Institute of Technology, Haifa, Israel; ^4^Rheumatology Unit, Tel Aviv University, Tel Aviv, Israel; ^5^Sackler School of Medicine, Tel Aviv University, Tel Aviv, Israel

**Keywords:** cardiolipin, systemic sclerosis, fibrosis, γδ T cells, zoledronate, phosphoantigens, interferon γ, interleukin-2

## Abstract

**Objectives:**

γδ T cells, a non-conventional innate lymphocyte subset containing cells that can be activated by lipids and phosphoantigens, are abnormally regulated in systemic sclerosis (SSc). To further evaluate the significance of this dysregulation, we compared how exposure to an autoantigenic lipid, cardiolipin (CL), during co-stimulation with an amino-bisphosphonate (zoledronate, zol), affects the activation and cytokine production of SSc and healthy control (HC) γδ T cells.

**Methods:**

Expression of CD25 on Vγ9^+^, Vδ1^+^, and total CD3^+^ T cells in cultured peripheral blood mononuclear cells (PBMCs), their binding of CD1d tetramers, and the effect of monoclonal antibody (mAb) blockade of CD1d were monitored by flow cytometry after 4 days of *in vitro* culture. Intracellular production of IFNγ and IL-4 was assessed after overnight culture.

**Results:**

Percentages of CD25^+^ among CD3^+^ and Vδ1^+^ T cells were elevated significantly in short-term cultured SSc PBMC compared to HC. In SSc but not HC, CL and zol, respectively, suppressed %CD25^+^ Vγ9^+^ and Vδ1^+^ T cells but, when combined, CL + zol significantly activated both subsets in HC and partially reversed inhibition by the individual reagents in SSc. Importantly, Vδ1^+^ T cells in both SSc and HC were highly reactive with lipid presenting CD1d tetramers, and a CD1d-blocking mAb decreased CL-induced enhancement of %SSc CD25^+^ Vδ1^+^ T cells in the presence of zol. %IFNγ^+^ cells among Vγ9^+^ T cells of SSc was lower than HC cultured in medium, CL, zol, or CL + zol, whereas %IFNγ^+^ Vδ1^+^ T cells was lower only in the presence of CL or CL + zol. %IL-4^+^ T cells were similar in SSc and HC in all conditions, with the exception of being increased in SSc Vγ9^+^ T cells in the presence of CL.

**Conclusion:**

Abnormal functional responses of γδ T cell subsets to stimulation by CL and phosphoantigens in SSc may contribute to fibrosis and immunosuppression, characteristics of this disease.

## Key Messages

Cardiolipin activates human Vδ1^+^ γδ T cells co-stimulated with zoledronate.
In systemic sclerosis, cardiolipin and zoledronate suppress the activation of Vγ9^+^ and Vδ1^+^ γδ T cells, respectively.
The CD1d molecule is involved in cardiolipin activation of Vδ1^+^ T cells in systemic sclerosis.

## Introduction

T cells are thought to be involved in the immune pathogenesis of systemic sclerosis (SSc) by their recognition of foreign or self-antigens that activate effector programmers leading to pathological fibrosis (1–3). While abnormal responses to conventional peptidic autoantigens have been identified, a few studies suggest that abnormal responses to lipid antigens and phosphoantigens may also play a role in the pathogenesis of SSc (4, 5). In this regard, γδ T cells expressing the Vδ1 gene segment in their T cell receptor (TCR) (Vδ1^+^ T cells) were found to be dysregulated in early and late SSc (6, 7). Thus, these cells were highly activated in the peripheral blood (PB) of patients, infiltrated the skin in early disease, and oligoclonally expanded in damaged internal organs (6, 7). Recent evidence indicates that a fraction of Vδ1^+^ TCR recognize lipid antigens presented by CD1d molecules expressed by antigen-presenting cells (APCs), suggesting a possible role for lipid antigens in the pathogenesis of SSc *via* effects on Vδ1^+^ T cells (8–10). In support of this, 10–20% of SSc patients have antibodies to cardiolipin (CL), a mitochondrial autolipid that is also present in microorganisms (11). Moreover, the T cell response to CL in a murine model of autoimmunity was independent of classical lipid responsive αβ TCR^+^ invariant natural killer T (iNKT) cells, suggesting that lipid reactive γδ T cells, rather than iNKT cells, may play a more critical role in disease-related autoimmune responses to CL (12). However, there is no available evidence to indicate that human γδ T cells in SSc recognize and respond to CL.

The second class of γδ T cells, characterized by expression of the Vγ9 gene in the γδ TCR (Vγ9^+^ γδ T cells), is also abnormally regulated in SSc. Thus, amino-bisphosphonate (ABP) compounds inhibit farnesyl pyrophosphatase, leading to increased levels of intracellular phosphoantigens [mainly isopentenyl pyrophosphate (IPP)] in APC that bind to and induce a conformational change in butyrophilin 3A1 (CD277) cell surface molecules on APC (13). This alteration is recognized by Vγ9^+^ TCR leading to Vγ9^+^ T cell activation (14, 15). In some previous publications, Vγ9^+^ T cells were shown to maintain functionality as cytotoxic effectors and cytokine producers in SSc and respond, albeit in a suppressed manner, to phosphoantigens, relative to healthy controls (HC) (5, 16). Other researchers, on the other hand, detected no significant difference between productivity of TNFα and IFNγ by γδ T cells in SSc patients and HC (17). Furthermore, intravenous treatment with zoledronate (zol), a potent ABP, adversely affected the clinical course in a SSc patient, suggesting that this reagent may have activated disease relevant pathogenic γδ T cells (18).

Indeed, the results presented in this article indicate for the first time, to our knowledge, that the functional programmes and activation of human Vδ1^+^ γδ T cells *in vitro* can be modulated by CL. Furthermore, activation is dependent on the CD1d lipid-presenting molecule and co-stimulation with zol. Importantly, the responses of γδ T cells to these stimuli differ between SSc and HC in a manner that could adversely affect immune responses and the fibrotic process characteristic of this devastating disease.

## Materials and Methods

This study was approved by the Institutional Review Board (Helsinki Committee) of the Sheba Medical Center, Ramat Gan, and Rambam Health Care Campus, Haifa, Israel. All patients and controls signed informed consent forms. Patients, described in Table 1, were treated in the Rheumatology Clinic at Sheba Medical Center in Ramat Gan, Israel, and at the B. Shine Rheumatology Unit at Rambam Health Care Campus in Haifa, Israel. All patients recruited for the study fulfilled criteria of the American College of Rheumatology for SSc (19). Controls included healthy donors from the hospital staff.

**Table 1:** Clinical characteristics of systemic sclerosis patients.

Patient	Age	Sex	ANA	SCI70	ACA	Organ involvement	Disease duration (years)	Subset	Treatment
1	58	F	+	−	+	GI, muscle	5	Limited	MTX, iloprost, IVIG
2	55	F	NA	NA	NA	Joints	0.5	Limited	Iloprost, HCQ
3	41	F	+	+	NA	Joints	4	Diffuse	MTX, iloprost, MabThera
4	67	F	NA	+		Kidney, lung, APS, cardiac	9	Diffuse	Iloprost, bosentan, prednisone
5	48	F	NA	NA	NA	Lung, GI, joints	15	Limited	Iloprost, bosentan, HCQ
6	83	F	+	−	NA	Lung	6	Limited	MTX, prednisone
7	44	M	+	+		Lung, pulmonary HTN	16	Limited	CYC
8	61	F	+	NA	NA		23	Limited	Iloprost, tracleer, plaquenil
9	58	M	+	−		Pericardium, joints, lungs	1	Limited	NA
10	42	M	+	NA	NA		20	Linear	HCQ, prednisone
11	60	M	+	NA	NA	Lung, pulmonary HTN, GI, cardiac	8	Diffuse	CYX, MTX, prednisone, bosentan, CCB
12	64	M	+	+		Lung, kidney, heart, joints	3	Diffuse	Rituximab, leflunomide, SSZ
13	48	F	+	+		Lung, digital ulcers, GI, joints	4	Limited	Prednisone, omeprazole, CYC, iloprost, MTX, bosentan
14	57	F	+	+		GI, digital ulcers	15	Limited	Prednisone, HCQ, iloprost, bosentan
15	47	F	+	−	+	GI, trigeminal neuralgia	6	Limited	Omeprazole
16	68	F	+	−	+	Digital ulcers	25	Limited	CCB
17	44	F	+	+		Digital ulcers	7	Diffuse	MMF, bosentan
18	58	F	+	+		Lungs, joints, GI	11	Diffuse	MMF, bosentan, prednisone, HCQ, omeprazole, iloprost
19	54	F	+	+		Lungs, joints, GI, digital ulcers, acroosteolysis	30	Diffuse	AZA, bosentan, prednisone, HCQ, omeprazole, iloprost

*ANA, antinuclear antibodies; APS, antiphospholipid syndrome; AZA, azathioprine; CCB, calcium channel blockers; CYC, cyclophosphamide; f, female; GI, gastrointestinal; HCQ, hydroxychloroquine; HTN, hypertension; IVIG, intravenous immunoglobulin; M, male; MTX, methotrexate; scl70, scleroderma 70 antibodies; SSZ, salazopyrin*.

### Reagents

OCH, an α-galactosylceramide analog with a truncated side chain that stimulates Th2-biased cytokine production in NKT, was obtained from the National Institutes of Health (NIH), USA, and stored at 0.2 mg/ml in an aqueous solution containing 0.5% Tween 20, 56 mg sucrose, and 7.5 mg histidine (20). CL (Sigma-Aldrich, Israel) was dissolved according to manufacturers’ instructions (stock solution 5 mg/ml) in methanol and stored at −2°C. The monoclonal antibodies (mAbs) for blocking experiments were anti-CD1d (Biolegend, USA) or IgG2b isotype control (Miltenyi Biotec, Germany). The mAbs for staining cell surface antigens included anti-CD3 APC (Tonbo Bioscience), anti-CD25 PE (BD Pharmingen, USA), anti-Vδ1-FITC (Endogen, Pierce, USA), and anti-Vγ9-FITC (Immunotech, USA). zol (Novartis, USA) and interleukin-2 (IL-2; Boeheringer–Mannheim, Germany) were used at the indicated concentrations.

### Isolation of PB Mononuclear Cells and Cell Culture

Peripheral blood mononuclear cells (PBMCs) were separated from 10 to 20 ml of heparinized blood on Lymphoprep gradients (AXIS-SHIEL, Oslo, Norway) by density centrifugation at 1,500 rpm as previously described (21). The mononuclear fraction was removed from the interface washed in phosphate-buffered saline (PBS) solution PH 7.4 and frozen in liquid nitrogen. Since our goal was to evaluate the effects of activation *in vitro*, and blood samples were relatively small, it was not always feasible to perform detailed cytometric analysis of freshly isolated cells. Thus, on the day of initiation of the experiments, freshly isolated PBMC of patients 1–12 in Table 1 and HC, per well placed at 2 × 10^5^ cells/well in 200 µl of final growth medium (FM) consisting of RPMI-1640 (Taassiot Biologiot, Beit Haemek Israell) supplemented with 2 mM l-glutamine, 10% fetal bovine serum, and penicillin–streptomycin solution (100 µg/ml) in 96-well round bottom culture plates. CL (2.5–10 µg/ml) or zol (2 µM) or IL-2 (100 IU/ml) or the mAb indicated above was added at the initiation of the cultures as indicated in individual experiments. After 4–5 days, cells were collected from the cultures, washed twice in PBS, and prepared for staining with mAbs for flow cytometry. In experiments involving analysis of cytokines, we used PBMC from patients 13–19 and corresponding HC samples that had been previously been frozen in liquid nitrogen.

### Flow Cytometry

Prior to analysis, cells were removed from culture, stained with trypan blue, and determined to be >95% viable by trypan blue exclusion. Cell staining procedures were as previously described (21). Lymphocytes were gated according to typical forward and side scatter plots using a BD FACS Calibur flow cytometer (Becton Dickinson, Mountain View CA, USA), and analysis of gated cells was performed using FlowJo software. Cells within designated gates (e.g., CD3^+^) were further analyzed. Percentage of cells expressing a marker (e.g., CD25) within the specific subset (e.g., CD3^+^, or Vγ9^+^ or Vδ1^+^) was calculated as follows: % dual positive cells/(% dual positive + % marker negative within subset); e.g., % CD25^+^Vγ9^+^ within subset = %CD25^+^Vγ9^+^/(%CD25^+^Vγ9^+^ + %CD25^−^Vγ9^+^).

### Cytokines

For intracellular cytokine staining, freshly isolated PBMC were cultured overnight alone, with CL (2.5 µg/ml), zol (2 µM), or both or with 50 ng/ml PMA (Sigma-Aldrich) and 1 mM ionomycin (Sigma-Aldrich), in the presence of brefeldin A (Sigma-Aldrich). After stimulation, cells were stained with APC-labeled mAb to Vδ1 (Miltenyi) or PC5-labeled mAb to Vγ9 (Beckmann Immunotech) or APC-labeled anti-CD3 (Tongo). The cells were fixed and permeabilized using a Cytofix/Cytoperm kit (eBioscience) and then stained using a mixture of FITC-labeled anti-IFNγ (eBioscience) and PE-labeled anti-IL-4 mAb (eBioscience). Intracellular cytokine staining was analyzed by flow cytometry.

### CD1 Tetramers Staining

CD1d tetramers bound to APC obtained from the NIH included: PBS-47 = a CD1d tetramer linked to an analog of α-galactosylceramide; CD1d^−^ OCH = a CD1d tetramer linked to an α-galactosylceramide analog with a truncated side chain; CD1d unbound = tetramers not linked to an exogenous antigen.

### Statistical Analysis

Results are expressed as mean ± 1 SEM. Statistical comparisons were drawn using a two-tailed Student’s *t*-test except where indicated using Excel Microsoft, Redmond WA software. A *p* value <0.05 was considered as statistically significant.

## Results

### Activation Status of γδ T Cell Subsets in Non-Stimulated Short-Term Cultures

**Figure 1:**
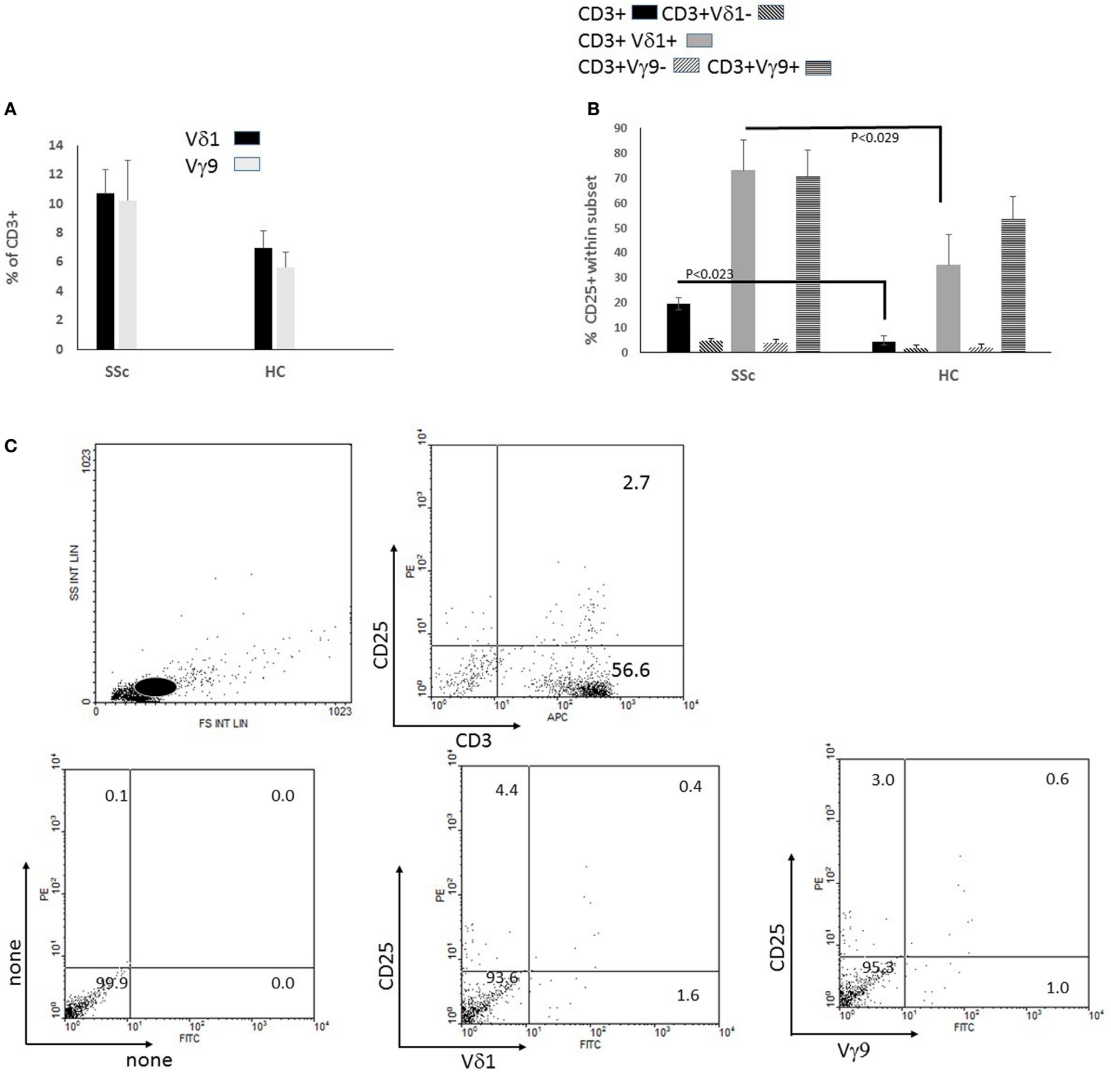
Percent of CD25^+^ cells in cultures of peripheral blood mononuclear cells (PBMCs) from patients with systemic sclerosis (SSc) vs healthy controls (HCs). PBMCs of 12 SSc patients (patients 1–12 in Table 1) and 8 HCs were incubated in medium alone for 4 days and then stained for flow cytometric analysis with fluorochrome-labeled monoclonal antibodies (mAbs) anti-CD25-PE, anti-Vδ1-FITC, anti-Vγ9-FITC, and anti-CD3-APC. **(A)** Data for the anti-Vδ1-FITC, anti-Vγ9-FITC staining of T cells in the cultures obtained by gating on CD3^+^ cells as shown in **(C)**. **(B)** Results for percent of cells within each of indicated subsets that were stained with anti-CD25-PE in **(B)** calculated as indicated in Section “Materials and Methods.” Bars represent the mean and SEMs. Significant differences between means by two-tailed Student *t*-test are indicated. **(C)** Dot plots represent on the upper left forward and side scattergram. The Lymphocyte gate is indicated with a white circle. On the upper right, dot plots of cells within the lymphocyte gate were stained with mAb to CD3 and CD25. The lower panel shows dot plots of cells within the gated CD3^+^ population unstained on the left, or with mAb to Vδ1 or Vγ9 bound to FITC, and CD25-PE as indicated.

γδT cells in SSc patients are highly activated *in vivo* to express HLA-DR (6, 22). We evaluated whether cell surface membrane expression of CD25, the IL-2 receptor α-chain, which is induced by TCR-mediated T cell activation, is likewise upregulated in SSc γδ T cells (23). Thus, we recorded, by flow cytometry, %Vδ1^+^ and Vγ9^+^ γδ T cells among the CD3^+^ lymphocytes in PBMC derived from SSc patients and HC and the percentage of CD25^+^ T cells within each subset, after brief *in vitro* culture in medium containing a low dose of IL-2 (100 IU/ml, FMIL-2). There were non-significant increases of %Vδ1^+^ and Vγ9^+^ T cells within the CD3^+^ population in cultures of PBMC derived from SSc patients (*n* = 12) relative to HC (*n* = 8) (Figure 1A). However, there was a significant increase of %CD25^+^ Vδ1^+^ and Vγ9^+^ T cells relative to the respective complementary Vδ1^−^ or Vγ9^−^ CD3^+^ populations in both SSc and HC cultures (Figure 1B). Moreover, % CD25^+^ within total CD3^+^ and also within the Vδ1^+^, but not Vγ9^+^ T cell populations, were significantly more highly represented in SSc than HC PBMC cultures (Figure 1B). The gating procedure for these analyses and an example of such analysis are shown in Figure 1C. These results suggested that T cells, consisting chiefly of αβ T cells, and the Vδ1^+^ T cell subset in particular maintain an abnormally highly activated status as reflected by CD25 expression after brief *in vitro* culture of SSc PBMC in the presence of IL-2.

### Effect of CL and zol on CD25 Expression on γδ T Cells Subsets in HC and SSc

**Figure 2:**
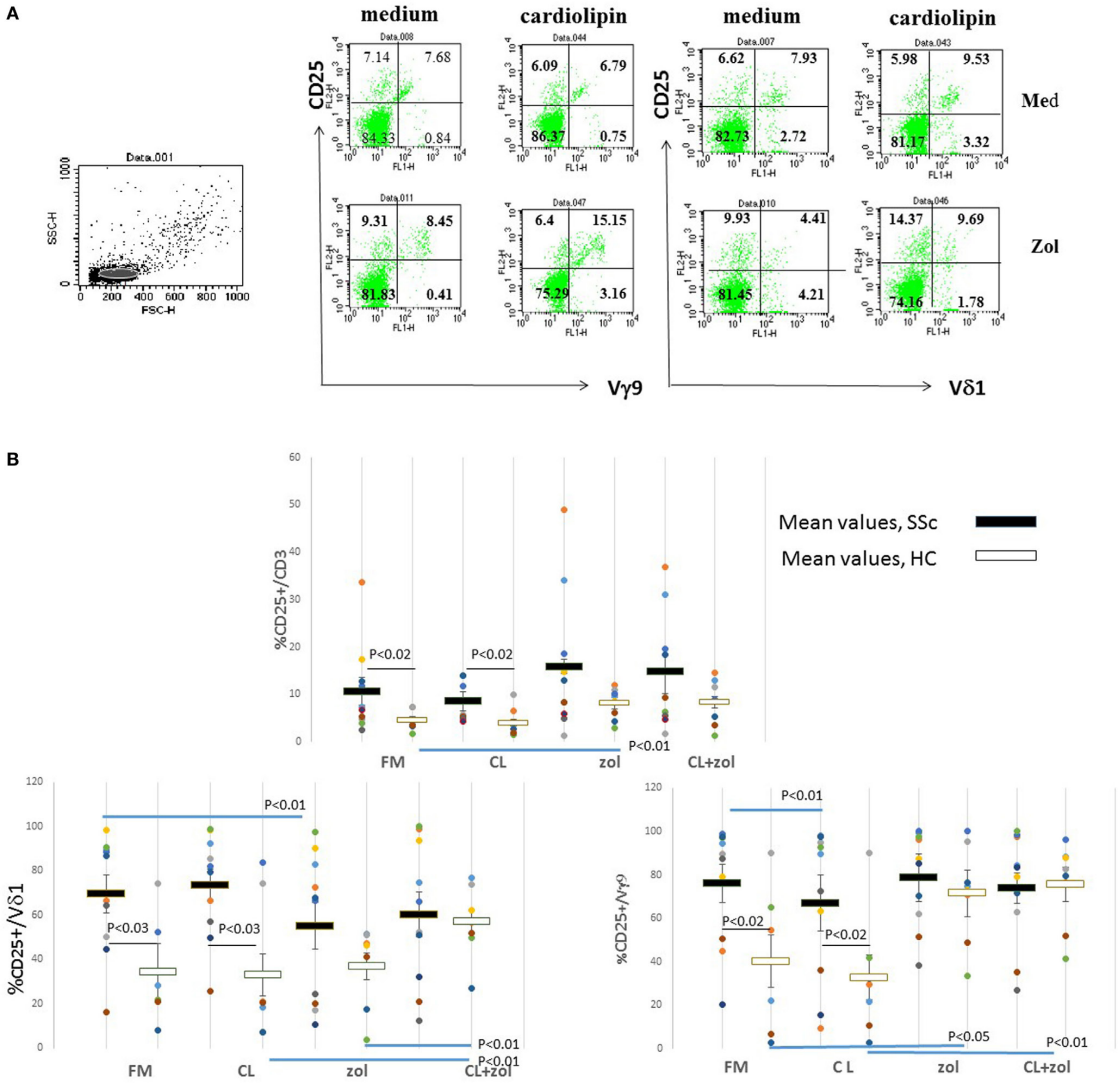
CD25 expression on γδ T cells in response to cardiolipin (CL) and zoledronate (zol). **(A)** Peripheral blood mononuclear cells (PBMCs) from a healthy control (HC) were incubated in medium with interleukin-2 (100 IU) alone or with added CL (2.5 µg/ml), either with or without zol (2 µM) for 4 days and analyzed by flow cytometry after staining with fluorochrome-labeled monoclonal antibodies (mAbs) anti-CD25-PE, anti-Vδ1-FITC, anti-Vγ9-FITC, and anti-CD3-APC. Forward and side scatter of the sampled cells are shown on the left; the lymphocyte population is highlighted in gray. On the right are shown dot plots of cells within total CD3^+^ population, representing both the CD3^+^Vδ1^−^ (containing most Vγ9^+^ T cells) and CD3^+^Vδ1^+^ populations after co-staining with mAb to CD25 and either Vδ1 or Vγ9. Percent of cells within indicated quadrants (set on the basis of non-stained cells) are shown. **(B)** PBMC from 10 systemic sclerosis (SSc; patients 1–10 Table 1) and 8 HC were cultured as mentioned in **(A)** and stained with mAbs to CD25, CD3, Vδ1, and Vγ9. Dots represent values for each culture of an individual. The mean percent and 1 SEM of CD3^+^ T cells, or of the indicated subsets, expressing CD25 in each of the culture conditions are indicated (medium with IL-2 = FM; medium with CL 2.5 µg/ml with IL-2 = CL; medium with IL-2 and zol 2 μM = z; zol with CL 2.5 μg/ml = CL + zol. Black horizontal bars represent mean for SSc and white for HC. *P* values calculated from two-tailed Student’s *t*-test comparing groups connected by horizontal lines are indicated.

Next, we evaluated how CL, which is a potential ligand for γδ and NKT cells, and zol, which activates Vγ9^+^ γδ T cells, influence %CD25^+^ T cells in the cultures (24–26). PBMCs were cultured in FMIL-2 or in FMIL-2 supplemented with zol (FMIL-2 zol), with or without CL, and expression of CD25 on subsets of T cells was evaluated by flow cytometry as indicated in an example using PBMC of a HC (Figure 2A). Neither CL (2.5 µg/ml) nor zol (2 µM) altered mean %CD25^+^CD3^+^ T cells in PBMC cultures of SSc patients (*n* = 10), whereas zol, but not CL, induced an increase of this subset in cultures of HC (*n* = 8) from 40.1 ± 12.1% to 71.6 ± 7.8% (*p* < 0.043; Figure 2B). In contrast, zol decreased the mean frequency of CD25^+^ cells in Vδ1^+^ T cells, and CL reduced it, in Vγ9^+^ T cells of SSc but not HC PBMC cultures. Furthermore, culture with combined CL + zol increased both mean %CD25^+^ Vδ1^+^ and Vγ9^+^ T cells in HC cultures significantly (Figure 2B). In cultures of SSc PBMC, however, CL + zol only partially abrogated the significant decrease induced by each reagent separately (Figure 2B).

**Figure 3:**
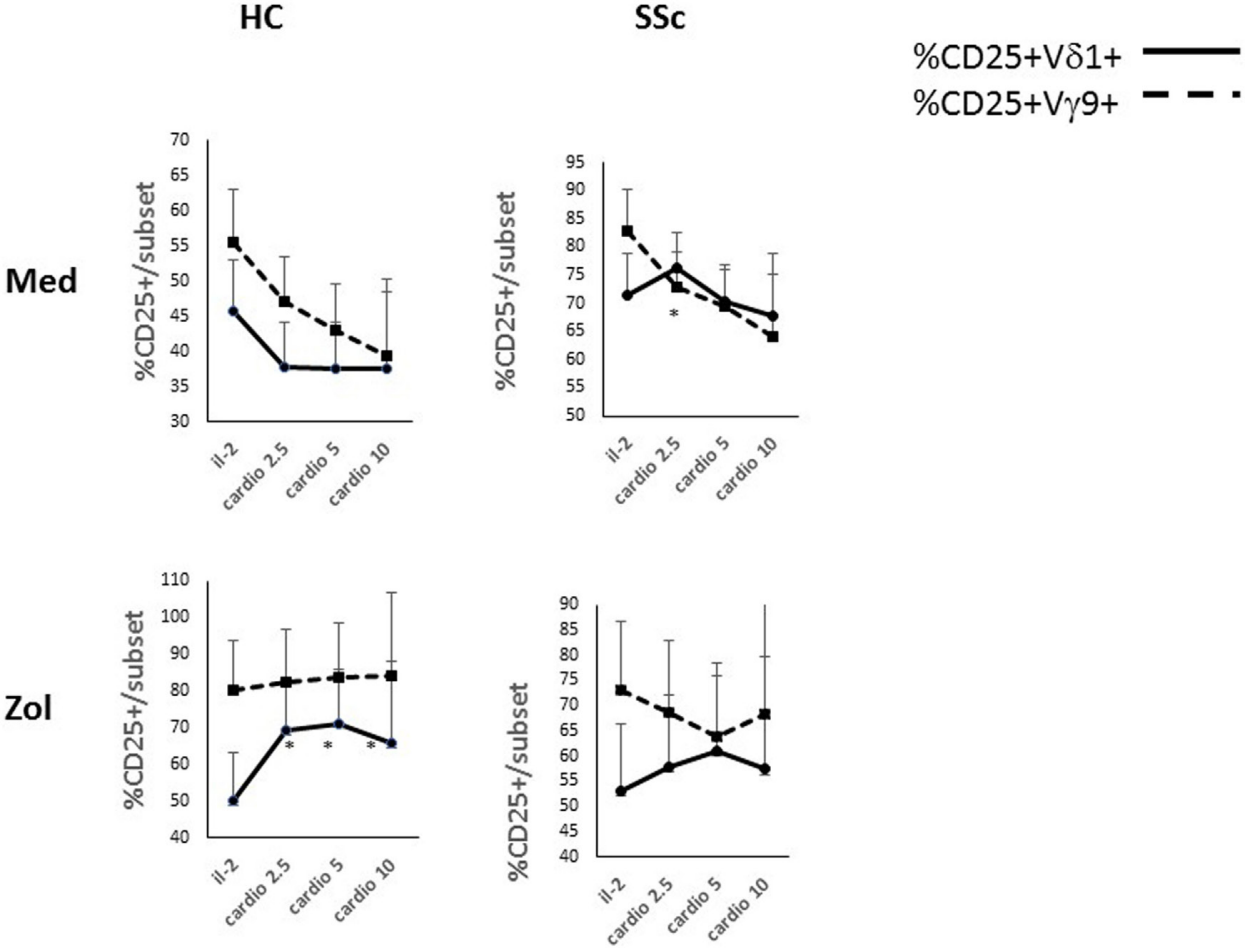
Dose effects of cardiolipin (CL) on cultured T cells in the presence and absence of zoledronate (zol). Dose response of % CD25^+^ Vδ1^+^ or Vγ9^+^ T cells as indicated (%CD25^+^ within a subset/total% of subset) after *in vitro* culture for 4 days in medium containing interleukin-2 (IL-2) 100 IU or, in addition, increasing concentration of CL (in microgram per milliliter) as indicated on the *x*-axis are shown. In parallel cultures, zol (2 µM) was added. Results represent mean and 1 SEM for cultures derived from three HCs and from five systemic sclerosis patients. Asterisks indicate a significant difference (*p* < 0.05) for comparison of the mean at the relevant concentration of CL to IL-2 alone.

We next examined the dose response of %CD25^+^ γδ T cell subsets in the cultures to increasing concentrations of CL (Figure 3). In HC PBMC cultured in FMIL-2, CL decreased mean %CD25^+^ Vδ1^+^ and Vγ9^+^ T cells non-significantly in a dose-dependent manner. In contrast, when added to FMIL-2 zol, it increased mean %CD25^+^ Vδ1^+^ T cells significantly and Vγ9^+^ T cells non-significantly. In SSc PBMC cultured in FMIL-2, % CD25^+^Vδ1^+^ TC increased slightly in the presence of 2.5 µg/ml of CL followed by a decrease at higher concentrations (Figure 3), along with a dose-dependent significant decrease of %CD25^+^Vγ9^+^ T cells. In FMIL-2 zol, %CD25^+^Vδ1^+^ and Vγ9^+^ T cells of SSc patients, respectively, increased or decreased in a CL dose-dependent manner, but in contrast to HC, the increase in the %CD25^+^Vδ1^+^ subset was not statistically significant. Taken together, these results suggest that, in the presence of zol and IL-2, CL induces the expression of CD25 on healthy human Vδ1^+^ T cells, whereas the response in SSc is blunted.

### CD1d Tetramer Binding to SSc and HC Vδ1^+^ T Cells

**Figure 4:**
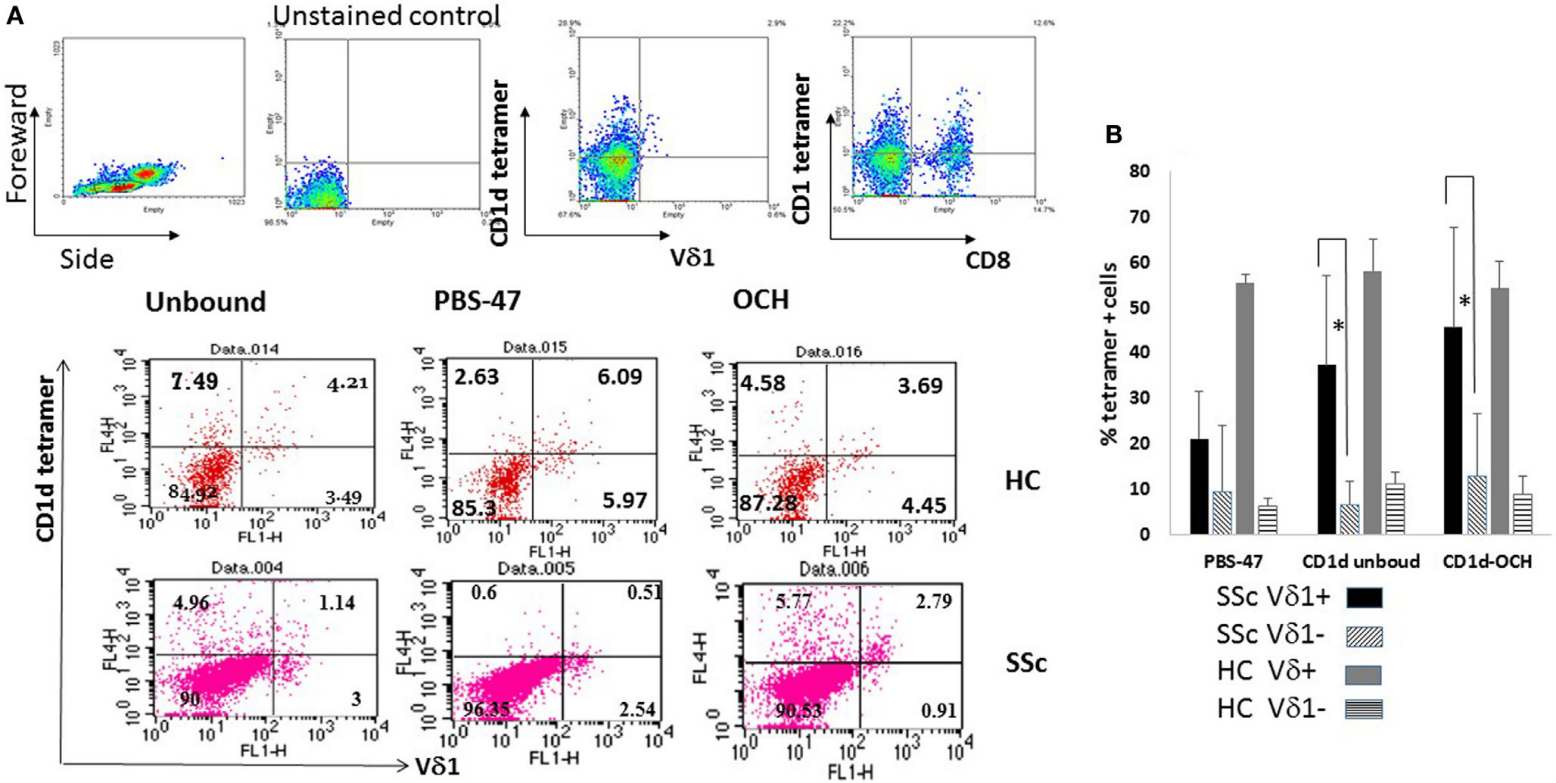
CD1d tetramer binding to cultured Vδ1^+^ T cells in systemic sclerosis (SSc) patients and healthy controls (HCs). **(A)** The upper panel shows gating strategy for analysis of CD1d tetramer binding using HC peripheral blood mononuclear cells (PBMCs) cultured in FM containing interleukin-2 (IL-2) 100 IU/ml for 6 days. Cells selected from the lymphocyte gate as indicated in the Forward/Side scattergram on the left were analyzed untouched or after staining with the CD1d unbound tetramer and monoclonal antibody (mAb) to either Vδ1 or CD8. The second and third panels represent flow cytometric dot plots of PBMC of a HC and SSc patient (patient 1; Table 1) cultured in FM containing IL-2 100 IU/ml for 6 days. The cells were stained with APC labeled CD1d tetramers (unbound, PBS-47 and bound to OCH) as indicated above the dot plots and Vδ1-FITC. Percent cells in quadrants set according to a non-stained control are shown. **(B)** Bars represent mean and 1 SEM of %Vδ1^+^ and Vδ1^−^ cultured cells binding the indicated tetramers in cultures as described in Figure 4A for five SSc patients and two HCs. * denotes *p* < 0.05 for adjacent indicated means.

We first determined whether Vδ1^+^ T cells of SSc patients can bind the tetramers of CD1d described in the Section “Materials and Methods.” Briefly cultured PBMC of SSc patients and HC were co-stained with mAb to Vδ1 and with fluorescently labeled native CD1d tetramers (CD1d unbound), CD1d tetramer complexed with a natural ligand, alpha galactosylceramide (PBS-47), or with an OCH derivative of this compound (CD1d-OCH) (27). By gating on lymphocytes, in a preliminary experiment as shown in the upper panel of Figure 4A, we ascertained the ability of the native CD1d tetramer to clearly detect cells within either Vδ1^+^ or Vδ1^−^ as well as on CD8^+^ and CD8^−^ cells in these brief cultures. In a further representative experiment, shown in the middle and lower panels of Figure 4A, 8–12% of the cells in HC cultures and 3–4% in an SSc patient-derived culture expressed Vδ1. Unbound, PBS-47 and CD1d-OCH stained both Vδ1^+^ T cells as well as Vδ1^−^ T cells in the cultures with the frequencies (relative to total cells in the cultures) indicated in each dot plot. Importantly, binding of all types of the tetramers was observed, even those not chemically attached to known lipids (CD1d unbound). Since CL binds to CD1d and is presented to human and murine iNKT cells and to murine γδ T cells (24, 25), it is possible that unbound CD1d tetramer, which is presumed to be loaded with lipid antigens endogenously expressed by the cells in which it is produced, includes a small proportion of CL-loaded CD1d tetramers.

Similar experiments of short-term PBMC cultures revealed that a mean of 21.2, 37.3, and 45.6% (*n* = 5, SSc) vs 55.5, 58.1, and 54.2% (*n* = 2, HC) of the Vδ1^+^ T cells, respectively, were co-stained by PBS-47, CD1d unbound, or CD1d-OCH tetramers (Figure 4B). Importantly, and as expected, due to known binding of CD1d to CD4^+^ and other NKT cells, we also detected a significant level of expression of CD1d tetramer-positive cells in the Vδ1^−^ populations of lymphocytes in the cultures, which included CD4^+^ and CD8^+^ T cells (Figure 4A and data not shown for CD4). In additional experiments, only inconsistent binding to Vγ9^+^ T cells was noted (not shown). However, an enhanced potential of briefly cultured Vδ1^+^ T cells of both SSc and HC to bind CD1d tetramers relative to Vδ1^−^ cells in the cultures was evident (*p* < 0.043, *p* < 0.042, and *p* < 0.082 for the respective tetramers for SSc patient; Figure 4B). These results suggested that the TCR of Vδ1^+^ T cells in SSc, similar to that of HC, bear the potential to recognize lipid antigens and possibly autoantigens presented by CD1d.

### Role of CD1d in Activation of SSc Vδ1^+^ T Cells in the Presence of CL and zol

**Figure 5:**
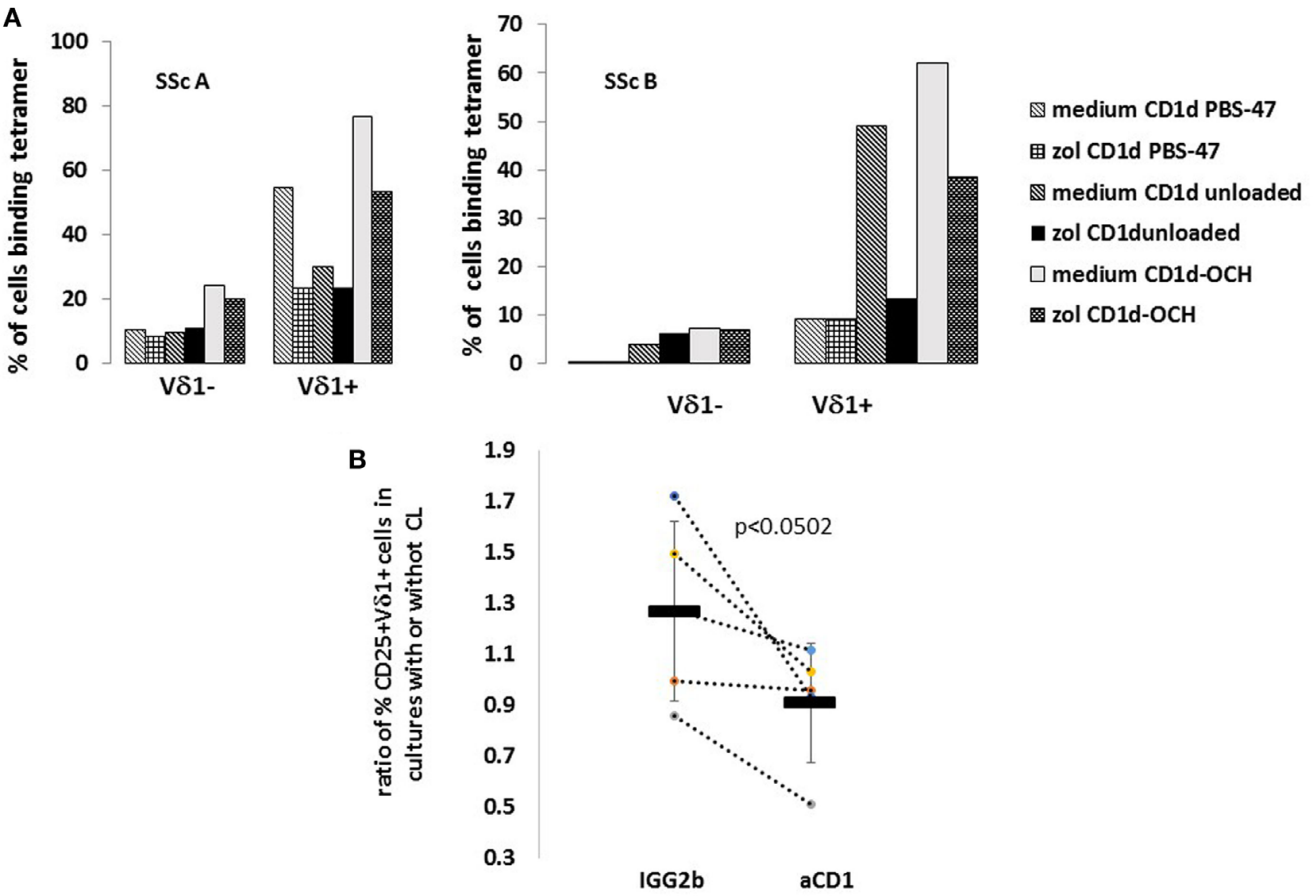
CD1d involvement in activation of Vδ1^+^ T cells by zoledronate (zol) and cardiolipin (CL) in systemic sclerosis (SSc) patients and healthy controls (HCs). **(A)** Peripheral blood mononuclear cells (PBMCs) from two SSc patients (SScA and SScB, patients 11 and 12; Table 1) were cultured in FMIL-2 alone (medium), or with 2 µM zol for 3 days, then co-stained with indicated antigen-presenting cell-labeled tetramers (CD1d unbound, CD1d PBS-47, and CD1d-OCH) and monoclonal antibody (mAb) to Vδ1-FITC, as described in figure. Bars show percent of cells binding the specific tetramers indicated in the legend within Vδ1^−^ and Vδ1^+^ cells in the cultures. **(B)** PBMC of five SSc patients (patients 8–12; Table 1) were cultured for 4 days with zol (2 µM) with or without CL (2.5 µg/ml), in the presence of a control non-binding mAb IgG2b or a mAb to CD1d. Subsequently, the expression of CD25 on the Vδ1^+^ T cells was assayed by flow cytometry and the %CD25^+^Vδ1^+^ calculated. The y axis represents the ratio of %CD25/Vδ1^+^ T cells in cultures with and without CL. Each dot represents an individual patient; results in the presence of the different mAb are connected by dotted lines. Means of values in the presence of control mAb and anti-CD1d are represented by horizontal bars; 1 SEM is indicated by vertical bars. *p* Value comparing means (Student’s one-tailed *t*-test) is indicated.

We then asked whether the observed decrease of %CD25^+^ Vδ1^+^ T cells in SSc-derived PBMC cultures in the presence of zol could be related to an effect of zol that leads to decreased binding of CD1d lipid antigen-presenting elements to the Vδ1^+^ T cells in SSc cultures. PBMCs from two SSc patients were cultured in FMIL-2 or FMIL-2 zol and after five days stained with CD1d tetramers. We detected decreased binding of the different CD1d tetramers to Vδ1^+^, but not to Vδ1^−^ T cells that had been cultured in FMIL-2 zol relative to those cultured in FMIL-2 alone (Figure 5A). Since CD25 expression is induced on T cells as a response to TCR binding of antigen-presenting molecules, these results suggest that zol-induced reduction of CD25 may be due to zol-mediated downregulation of CD1d binding to the Vδ1^+^ γδ TCR.

To confirm that CD1d-mediated interactions plays a role in activation of SSc Vδ1^+^, we tested the ability of mAb to CD1d to reverse the low level activation of %CD25^+^ Vδ1^+^ T cells induced by CL in the presence of zol (Figure 3). PBMC of SSc patients (*n* = 5) were cultured in medium containing zol with or without added 2.5 µg/ml CL, in the presence of either an irrelevant isotype control mAb or a blocking mAb to CD1d. As illustrated in Figure 5B, in the presence of the isotype control mAb, %CD25^+^Vδ1^+^ T cells increased 25% in CL-containing cultures compared to cultures without CL. In contrast, in the presence of mAb to CD1d, %CD25^+^Vδ1^+^ T cells decreased 9% relative to cultures without CL (*p* < 0.0502). These results suggest that CL is recognized by CD1d, which may lead to enhancement of %CD25^+^Vδ1^+^ T cells.

### IFNγ and IL-4 Production by SSc and HC Vδ1^+^, Vγ9^+^, and Total T Cells

**Figure 6:**
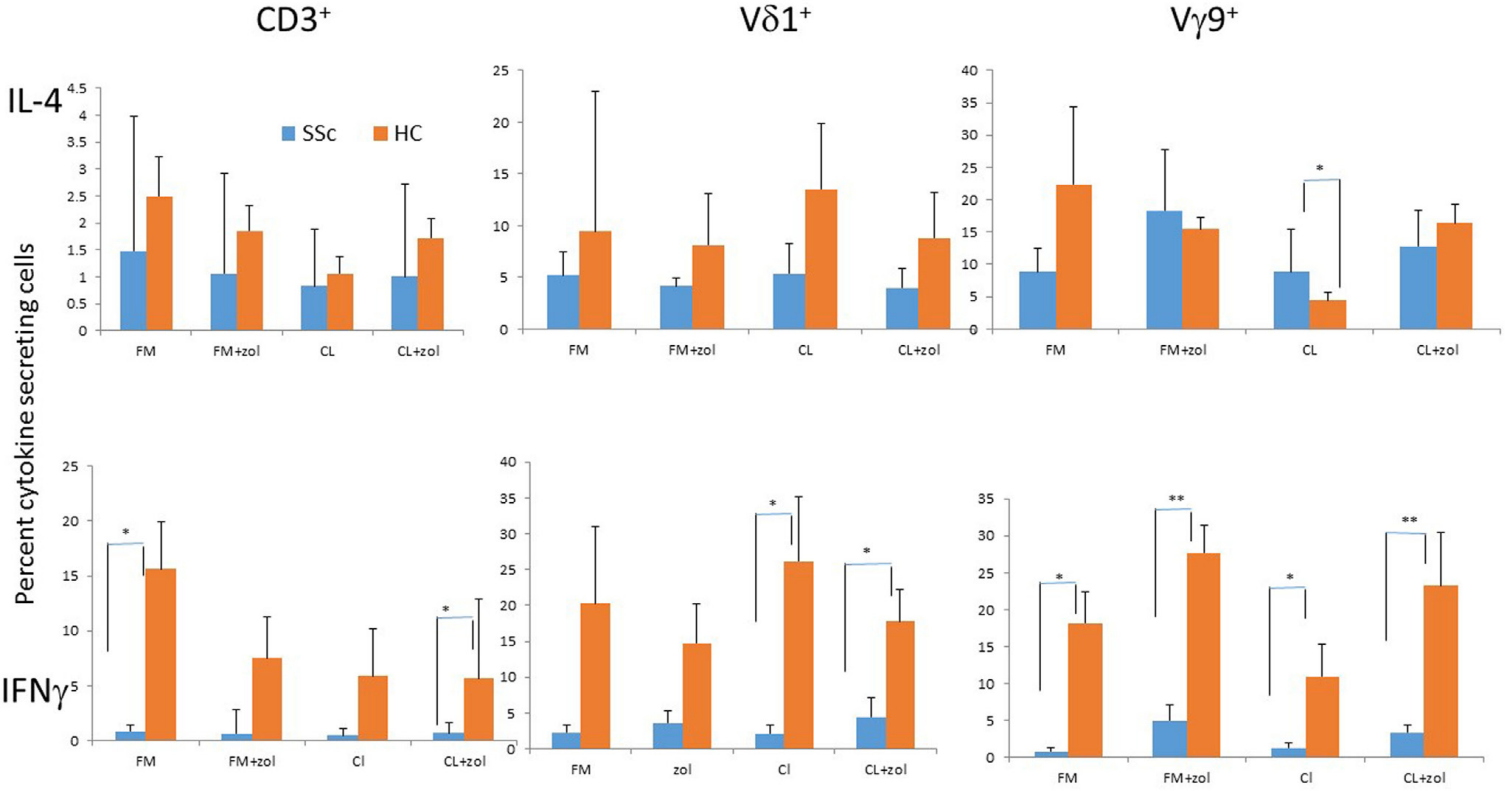
Cytokine production by systemic sclerosis (SSc) patients and healthy controls (HCs) T cells. Peripheral blood mononuclear cells from seven SSc patients (patients 13–19; Table 1) and five HC were cultured overnight in medium alone (FM), cardiolipin (CL), zoledronate (zol), or both (FM + zl or CL + zol). Subsequently, cell surface membranes were stained with monoclonal antibody (mAb) to CD3, Vγ9, or Vδ1, and cells were stained intracellularly with mAb to IL-4 (upper panel) or IFNγ. Bars indicate the mean ± 1 SEM of %cytokine^+^ cells for the subsets as indicated. *p < 0.05 and **p < 0.005 for comparison of adjacent SSc and HC mean values, respectively.

IFNγ and IL-4, respectively, oppose or support fibrosis, a central pathological feature of SSc. We previously reported decreased expression of IFNγ by SSc relative to HC Vγ9^+^ γδ T cells in response to zol, and also secretion of IL-4 by Vδ1^+^ T cells that was induced by zol in an SSc patient (18, 20). Here, we analyzed by flow cytometry (see Materials and Methods) how CL and zol affect intracellular cytokine production by the T cells from SSc patients and HC, after brief *in vitro* activation. The mean % IL-4^+^ cells among CD3^+^, Vγ9^+^, and Vδ1^+^ T cells in PBMC of SSc (*n* = 7, patients 13–19 in Table 1) and HC (*n* = 5) in the presence of non-TCR-dependent stimulation (PMA and ionomycin) were virtually identical (respectively, 28.2 ± 22.2 vs 35.0 ± 8.22, *p* < 0.75, 34.0 ± 9.18 vs 32.1 ± 9.18, *p* < 0.49, 15.6 ± 4.7 vs 16.5 ± 8.7, *p* < 0.91). In contrast, the frequency of IFNγ-secreting cells trended to be uniformly yet non-significantly lower in SSc (28.4 ± 6.97 vs 48.96 ± 9.05, *p* < 0.09, 47.8 ± 11.37 vs 75.9 ± 2.34, *p* < 0.083, 40.4 ± 9.62 vs 71.9 ± 9.68, *p* < 0.058). Contrasting with the non-significant differences in the presence of PMA + ionomycin, % IFNγ^+^ CD3^+^ T cells were significantly lower in SSc PBMC in medium alone or in the presence of both CL and zol (Figure 6). %IFNγ^+^ Vγ9^+^ T cells were uniformly and significantly reduced in SSc PBMC in all culture conditions, including medium alone and medium with CL, zol, or both reagents. In contrast, %IFNγ^+^ Vδ1^+^ T cells in SSc PBMC was lower in medium and zol than in HC but significantly only in the presence of CL or CL + zol. On the other hand, while there was no significant difference in % IL-4-secreting cells among CD3^+^ and Vδ1^+^ T cells of SSc and HC in any of the culture conditions, % IL-4 ^+^Vγ9^+^ T cells in SSc cultures were significantly increased relative to HC but only in the presence of CL.

Taken together, these results indicate that T cells in SSc retain a reduced potential to secrete the antifibrotic IFNγ. Importantly, the Vγ9^+^ T cell subset appeared to be most highly significantly suppressed in its ability to secrete IFNγ relative to HC, whereas the lower production of this cytokine in the Vδ1^+^ T cell subset of SSc patients strictly required CL. Moreover, CL increased secretion of the profibrotic IL-4 cytokine by the Vγ9^+^ T cell subset in SSc relative to HC. Together these results suggest that exposure to CL may induce a profibrotic phenotype within the γδ T cell subset in SSc.

## Discussion

Dysregulated T cell functions are thought to contribute to the profibrotic phenotype in SSc (28). Here, we focused on the role of a self-lipid antigen, CL, a putative autoantigen, and of an inducer of phospho-antigens (zol), in the regulation of activation and cytokine secretion by γδ T cells in SSc. Despite the small sample number of patients studied and large individual variability, our results revealed a statistically significant *a priori* heightened state of activation of Vδ1^+^ γδ T cells in SSc manifested by increased expression of CD25 in short-term cultures absent of any additional external antigenic stimulation. Next, we found that CL exerted several statistically significant effects, including:

- Decreased CD25 expression of the Vγ9^+^ T cells of SSc patients but not of HC.
- In conjunction with co-stimulation by a Vγ9^+^ T cell activator, zol, significant enhancement of CD25 expression in HC and, to a lesser degree, in SSc Vδ1^+^ T cells.
- Decreased secretion of IFNγ by SSc Vδ1^+^ T cells relative to HC while inducing an increase, relative to HC, in the secretion of IL-4 by patient Vγ9^+^ T cells.

Furthermore, although we have not directly shown binding of a CL-bound CD1d tetramer to SSc Vδ1^+^ T cells, the involvement of CD1d lipid-presenting molecules in CL-induced SSc-specific perturbations of γδ T cell subset activation was suggested by the finding that CL + zol activation of SSc Vδ1^+^ T cells was inhibited by a mAb to CD1d. Together, these *in vitro* findings suggest that CL could contribute to the immune dysregulation in SSc in a direction that could enhance fibrosis by cytokine dysregulation. In addition, cross-interactions between CL activated SSc Vδ1^+^ T cells and phosphoantigen activated Vγ9^+^ T cells may cross-modulate activation of the respective subsets.

CD25 is a marker of the state activation of T cell subsets induced by interactions of the TCR with its ligands (23). An increased CD25 expression on Vδ1^+^ T cells in short-term cultures of SSc (relative to HC) in the absence of any additional triggers other than low-dose IL-2, suggests that enhanced activation of these cells *in vivo* as previously demonstrated by Giacomelli et al., which is presumably triggered by autoantigens, is maintained *in vitro* (Figure 1B) (6). Since a subset of Vδ1^+^ T cells can recognize lipids, these results suggest that an abnormal response to autoantigenic or foreign lipids could be involved in the increased level of activation found in SSc Vδ1^+^ T cells. The autoantigen we chose to study in this context, CL, is known to elicit a humoral immune response during infections, including hepatitis, HIV, *Treponema pallidum*, and *Coxiella burnetii* as well as in a variety of autoimmune diseases (29–31). More specifically, in localized SSc, antibodies to CL are highly prevalent, suggesting a link to fibrotic processes in this disease even in the absence of other systemic features (11). Abnormal levels and structure of CL play a role in idiopathic pulmonary fibrosis, as well as in bleomycin-induced fibrosis, which is often used as a murine model for SSc (32). Thus, lysocardiolipin acyltransferase, a CL-remodeling enzyme, protects against lung fibrosis induced by bleomycin, suggesting a critical role for CL in the fibrotic process. This is thought to be related to correction of bleomycin-induced decrease of the CL level and of the unsaturated to saturated fatty acid ratio and unsaturation index in the CL. These findings thus suggest a pivotal pathogenic role for CL in SSc (32).

Moreover, CL reactive γδ T cells have been described in the T cell repertoire of rodents, suggesting that such cells might exist in the human γδ compartment as well (26). Although it is known that CL binds to human CD1d tetramers that are recognized by the TCR of human Vδ1^+^ T cells and NKT cells, the data presented here are the first to provide evidence that this autoantigen indeed plays a role in human Vδ1^+^ T cell activation (24). Moreover, our data suggest, for the first time, that CL elicits different responses in HC and in SSc γδ T cells cultured *in vitro*.

**Figure 7:**
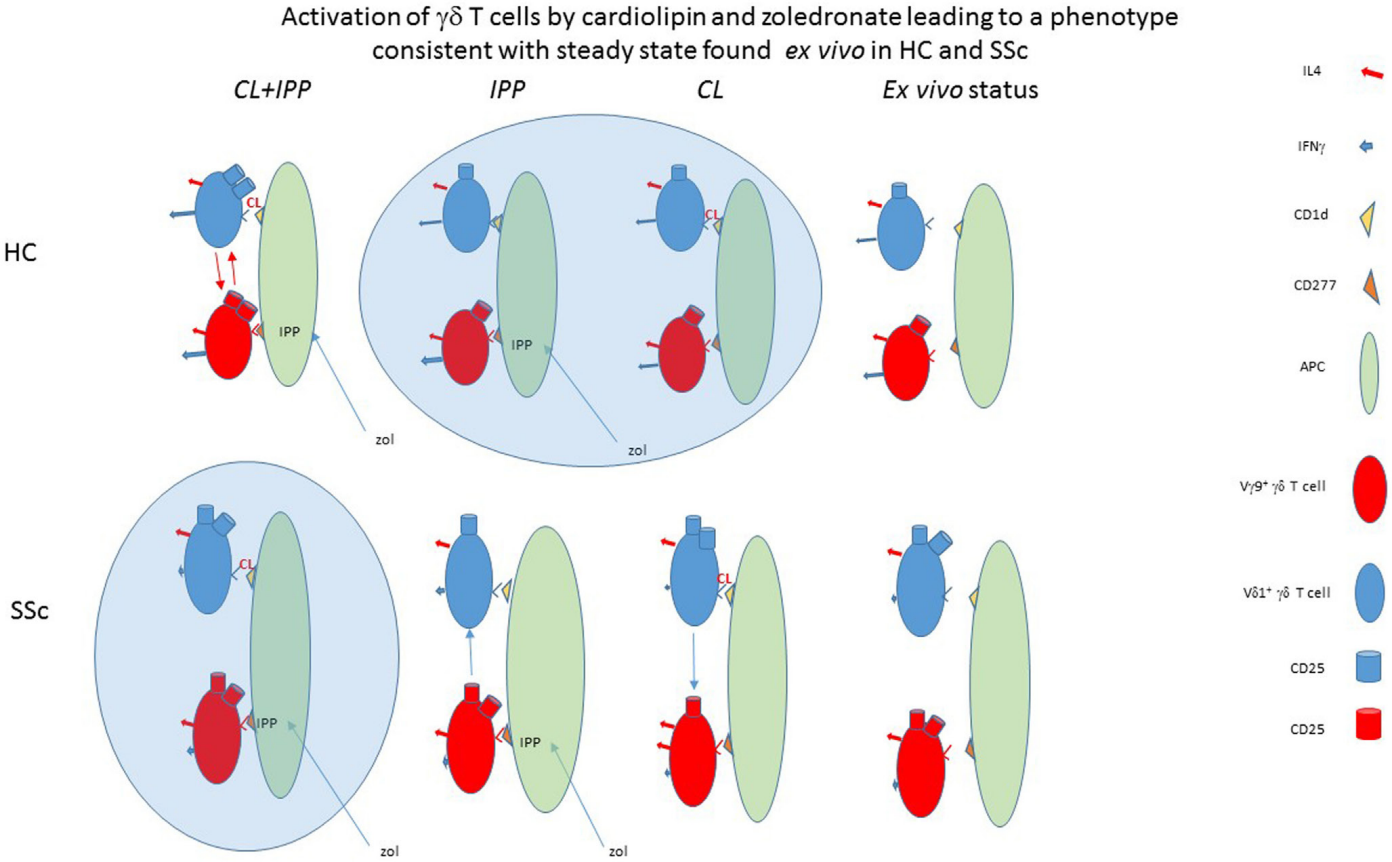
Model of differential cardiolipin (CL) and zoledronate (zol) effects in systemic sclerosis (SSc) patients and healthy controls (HC). The cartoon shows Vδ1^+^ or Vγ9^+^ T cells interacting with an APC bearing CD1d and CD277, in the presence or absence of CL and/or isopentenyl pyrophosphate (IPP) induced by zol, respectively, presented by CD1d or CD277. Response of the antigen-triggered γδ T cells to antigen stimulation is reflected by expression of CD25 and release of cytokines. Magnitude of response is represented by the number of CD25 symbols or size of the cytokine arrow, using HC as the standard. The phenotype of *ex vivo* cultured cells without any stimulation is depicted on the right as indicated. As seen, the *ex vivo* non-stimulated phenotype is consistent with concurrent dual stimulation (CL and zol) of the Vδ1^+^ and Vγ9^+^ T cells in SSc and with each separately in HC.

Interestingly, we found that the enhancement of the percentage of HC Vδ1^+^ T cells expressing CD25 induced by CL was dependent on co-stimulation of PBMC cultures with zol. These results suggest that CL activation of Vδ1^+^ T cells *in vitro* requires simultaneous signals that can be supplied by phosphoantigen-triggered Vγ9^+^ T cells (Figures 2, 3 and 7). Zolendronate is taken up by monocytes, increasing production of intracellular phosphoantigens, mainly IPP, in the mevalonate pathway by inhibition of farnesyl pyrophosphate synthase (33). IPP binds intracellularly to butyrophilin 3A1 (CD277) expressed on APCs, which triggers a cognitive TCR-mediated activation of the Vγ9^+^ γδ T cells to secrete cytokines, express activation markers, and also, remarkably, to differentiate into APCs (14, 33–35). A recent study demonstrated that this activation may also result in the expression of CD1d on the cell surface membrane of the Vγ9^+^ T cells *via* trogocytosis of CD1d-expressing B cell and activated macrophage membranes (36). Thus, the “helper” role of zol in terms of activation of the Vδ1^+^ T cell subset of HC in the presence of CL could be related to its effects on cytokines, induction of co-stimulatory molecules on APC expressing CD1d, or *de novo* CD1d-dependent presentation of CL by the Vγ9^+^ T cells themselves. To resolve the critical question of cross-regulation of the γδ T cell subsets and how APC and other CL-responsive cells may be involved, future experiments using isolated populations and studies to investigate the role of direct cell contact as opposed to that of soluble mediators are required. Nevertheless, irrespective of the mechanisms involved, we postulate that the *a priori* elevated expression of CD25 on SSc Vδ1^+^ T cells *ex vivo* (Figure 1) may have been elicited *in vivo* by exposure to CL in the presence of activated Vγ9^+^ T cells (Figure 7).

Plausible scenarios leading to activated SSc Vδ1^+^ T cells *in vivo* include viral infections, intracellular bacterial infections, and malignant transformation, all of which are associated with overproduction of IPP (37). Vγ9^+^ T cells activated by IPP induce apoptosis of the affected cells, exposing CL on the surface membrane, which may be taken up by Vγ9^+^ T cells and dendritic cells for CD1d-mediated presentation to responding iNKT and Vδ1^+^ T cells (5, 36, 38). Since CL enhanced CD25 expression by Vδ1^+^ T cells in SSc during zol co-stimulation was inhibited by a blocking mAb to CD1d, we suggest that activation of SSc Vδ1^+^ T cells in the presence of co-stimulation with zol is dependent on Vδ1^+^ TCR interactions with CD1d on the APC.

It is noteworthy that %CD25^+^ Vδ1^+^ T cells of SSc patients decreased in the presence of co-stimulation with zol in the absence of CL, suggesting that, in the absence of the autoantigen CL, SSc IPP-activated Vγ9^+^ T cells subset may suppress Vδ1^+^ T cell activation. Interestingly in this regard, whereas the direct IPP Vγ9^+^ T cell stimulator can quite efficiently activate SSc Vγ9^+^ T cells, zol is quite inefficient in this regard, consistent with our previous report (4, 5) Our data suggest that zol-induced decrease of CD25 expression might involve decreased binding of CD1d (presumably *via* their TCR) to the Vδ1^+^ T cells, by an unknown underlying mechanism (Figure 5). Conversely, Vγ9^+^ T cell activation manifested by CD25 expression was impeded by CL in the absence of zol, in SSc specifically. CL inhibition of Vγ9^+^ T cells in SSc could be mediated by Vδ1^+^ CD1d-CL complex reactive cells, since their partial inhibition in the presence of both CL and zol was reversed by anti-CD1d (data not shown). One possibility is that decreased CD25 expression entails alterations of cytokine secretion since CL, even in the absence of zol, decreased IFNγ in Vδ1^+^ T cells and enhanced IL-4 in Vγ9^+^ T cells relative to what is found in HC (Figure 6). Further studies of how interactions between activated γδ T cell subsets in HC and SSc are regulated by antigens and autoantigens could shed new light on dysregulated immune responses in SSc. It is important to note, however, that our patient population was not homogeneous, and most were treated with immunosuppressive drugs, which might have affected the outcome of these experiments (Table 1). Interestingly in this regard, our previous study failed to reveal any consistent effect of a potent immunosuppressive agent, cyclophosphamide, on percentages of Vγ9^+^ or Vδ1^+^ T cells among CD3^+^ T cells in a small number of SSc patients (4, 5). Furthermore, our data presented in Figure 1 show that both CD3^+^ and Vδ1^+^ and, to a lesser extent, Vγ9^+^ T cells showed an increased, rather than decreased, proportion of CD25^+^ T cells in the absence of antigenic stimulation. Thus, it is unlikely that the immunosuppressant reagents used in the patients might have significantly affected the results of antigen stimulation *in vitro*. Nevertheless, further experiments of untreated patients will be necessary to rigorously exclude the effects of medications used in SSc on the γδ T cell response to zol and CL.

A recent report indicated that CD161^+^ Vδ1^+^ T cells secreting diminished amounts of the antifibrotic cytokine IFNγ are expanded in SSc patients (39). Our results suggest that exposure to a lipid autoantigen such as CL plays an active role in suppression of IFNγ production in SSc Vδ1^+^ T cells (Figure 6). In addition, in the experimental conditions used in this article, Vγ9^+^ T cells of SSc patients also produced less IFNγ than HC, consistent with our previous independent experiments, and this relative decrease was maintained in the presence of CL (16). On the other hand, although IL-4 was not significantly different in SSc and HC T cells and was not suppressed by CL or zol in SSc relative to HC, % IL-4 secreting Vγ9^+^ T cells surprisingly increased (relative to HC) in the presence of CL. The mechanism underlying this shift is not clear, but could hypothetically also be mediated by CL reactive Vδ1^+^ T cells as postulated in the cartoon, as a consequence of the reduction of IFNγ induced in these cells by CL (Figure 7). In all, the abnormal responses of the γδ T cell subset in the SSc population to CL appear to shift the balance of cytokines in SSc, to a more profibrotic profile *via* IL-4 increase and IFNγ decrease, and to a more immune suppressive one via decreased activation of Vγ9^+^ T cell responses to phosphoantigens. Thus, we speculate that, in the setting of infection or other IPP-inducing cellular stresses such as cancer, CL-induced activation of IL-4-producing γδ T cells in SSc, together with a decrease of IFNγ-producing γδ T cells, may contribute to organ fibrosis. This idea is based on the known antifibrotic effect of IFNγ, vs the profibrotic effects of IL-4 (40). Interestingly, these changes may also have impacted the vascular aspects of SSc, as occurred in a previously described patient whose disease dramatically deteriorated after intravenous zol therapy (18, 41). Because the increase of IL-4 detected in these *in vitro* experiments was relatively small, further work to determine the role of IL-4 vs IFNγ-producing γδ T cells *in vivo* in biopsies of afflicted tissues from SSc patients will be required. Furthermore, it remains to be determined whether the effect of CL is able to overcome the anti-fibrotic effect (mediated by cytotoxicity against fibroblasts) of IPP-activated Vγ9^+^ T cells (4, 5).

In summary, effects induced by CL and possibly by other autoantigenic lipids or foreign lipid antigens, may have important implications for the dysregulation of γδ T cell subsets and, perhaps, for the pathogenesis of the fibrotic phenotype in SSc. Further experiments, including isolation of different mononuclear cells subsets, including those other than the γδ T cell subset, and studies of the roles of cell contact and soluble mediators are warranted to fully understand the role of autoantigenic lipids and phosphoantigens in SSc.

## Ethics Statement

The study was approved by the Institutional Review Board (Helsinki Committee) of the Sheba Medical Center, Ramat Gan, and Rambam Health Care Campus, Haifa, Israel.

## Author Contributions

HS: substantial contributions to the conception or design of the work; the acquisition, analysis, and interpretation of data for the work; drafting the work; and final approval of the version to be published. JH: substantial contributions to the conception or design of the work; analysis and interpretation of data for the work; and final approval of the version to be published. PF: substantial contributions to the conception or design of the work; analysis and interpretation of data for the work; and final approval of the version to be published. AB-G: substantial contributions to the conception or design of the work; the acquisition, analysis, and interpretation of data for the work; patients’ care and sampling; drafting the work and revising it critically for important intellectual content; and final approval of the version to be published. YB-M: interpretation of data for the work; patients’ care and sampling; drafting the work and revising it critically for important intellectual content; and final approval of the version to be published. IB: substantial contributions to the conception and design of the work; the acquisition, analysis, and interpretation of data for the work; patients’ care and sampling; drafting the work and revising it critically for important intellectual content; final approval of the version to be published; and agreement to be accountable for all aspects of the work in ensuring that questions related to the accuracy or integrity of any part of the work are appropriately investigated and resolved.

## Conflict of Interest Statement

IB is an inventor of a patent regarding *ex vivo* expansion of γδ T cells for treatment of diseases. All other authors declare that the research was conducted in the absence of any commercial or financial relationships that could be construed as a potential conflict of interest. The reviewer GG and handling Editor declared their shared affiliation.
